# Light-field microscopy with correlated beams for high-resolution volumetric imaging

**DOI:** 10.1038/s41598-022-21240-1

**Published:** 2022-10-07

**Authors:** Gianlorenzo Massaro, Davide Giannella, Alessio Scagliola, Francesco Di Lena, Giuliano Scarcelli, Augusto Garuccio, Francesco V. Pepe, Milena D’Angelo

**Affiliations:** 1 Dipartimento Interateneo di Fisica, Università degli Studi di Bari “Aldo Moro”, 70126 Bari, Italy; 2 Istituto Nazionale di Fisica Nucleare, Sezione di Bari, 70125 Bari, Italy; 3 Fischell Department of Bioengineering, University of Maryland, College Park, MD 20742 USA

**Keywords:** Optics and photonics, Applied optics, Optical physics, Optical techniques

## Abstract

Light-field microscopy represents a promising solution for microscopic volumetric imaging, thanks to its capability to encode information on multiple planes in a single acquisition. This is achieved through its peculiar simultaneous capture of information on light spatial distribution and propagation direction. However, state-of-the-art light-field microscopes suffer from a detrimental loss of spatial resolution compared to standard microscopes. In this article, we experimentally demonstrate the working principle of a new scheme, called Correlation Light-field Microscopy (CLM), where the correlation between two light beams is exploited to achieve volumetric imaging with a resolution that is only limited by diffraction. In CLM, a *correlation image* is obtained by measuring intensity correlations between a large number of pairs of ultra-short frames; each pair of frames is illuminated by the two correlated beams, and is exposed for a time comparable with the source coherence time. We experimentally show the capability of CLM to recover the information contained in out-of-focus planes within three-dimensional test targets and biomedical phantoms. In particular, we demonstrate the improvement of the depth of field enabled by CLM with respect to a conventional microscope characterized by the same resolution. Moreover, the multiple perspectives contained in a single *correlation image* enable reconstructing over 50 distinguishable transverse planes within a 1 mm^3^ sample.

## Introduction

Rapid imaging of three dimensional samples at the diffraction limit is a long-standing challenge of microscopy^1^. Many attempts are being made to address the need for rapid imaging of large volumes, with acquisition speed sufficient to analyze dynamic biological processes, all leading to different kinds of tradeoff. Progresses in this field include depth focal scanning with tunable lenses^2,3^, light-sheet illumination^4^, also employing non-diffracting beams^5–10^, fast STED^11^, fast two-photon microscopy^12^, and multi-focus multiplexing^13^. Software techniques such as compressive sensing and computational microscopy^14^ are also employed to improve performances. In this perspective, light-field microscopy is among the most promising techniques. By detecting both the spatial distribution and the propagation direction of light, in a single exposure, light-field imaging has introduced the possibility to refocus out-of-focus parts of three-dimensional samples, in post-processing. The depth of field (DOF) within the imaged volume can thus be extended by stacking refocused planes at different distances^15–20^. However, in its traditional implementation, light-field imaging is affected by the fundamental barrier imposed by the resolution versus DOF compromise. In the microscopic domain, this tradeoff is particularly suboptimal, since the required high resolution strongly limits the DOF, making it necessary to perform multiple scanning to characterize a thick sample^21^. In microscopy applications, light-field microscopy could offer a solution to the bottlenecks of long acquisition times, typical of scanning approaches, and the unbearably large amount of data, typical of multi-focus multiplexing. However, its widespread application has been stifled by the degraded resolution, far away from the diffraction limit^16,18^. Nevertheless, fostered by the development of image analysis tools and deconvolution algorithms that provide a partial recovery of resolution^22–24^, light-field imaging has shown its potential in neuroscience applications, where it was employed to analyze firing neurons in large areas^25^. A miniaturized version of a light-field microscope was also recently employed to enable microscopy in freely moving mice^26^.

In this article, we provide the experimental demonstration of a novel method to perform light-field microscopy with *diffraction-limited resolution*, discussing its realization and testbed applications. The new technique, capable of beating the classical microscopy limits by exploiting the statistical properties of light^27–32^, employs the working principle of Correlation Plenoptic Imaging (CPI)^33–38^, in which light-field imaging is performed at the diffraction limit by measuring correlations between intensity fluctuations at two disjoint detectors^39–43^. Previous CPI architectures were limited to bright-field operation relying on mask objects. Here, we design a CPI architecture suitable for different microscopy modalities (fluorescence, polarization, dark-field) that are critical for biological applications. To this end, the sample is illuminated with the whole light beam from a chaotic light source^44^, rather than by just one beam out of a correlated beam pair^33,36,37^. This enables imaging self-emitting, scattering and diffusive samples, as well as performing birefringent imaging, without sacrificing the retrieved correlation. Further advantages of the proposed Correlation Light-field Microscopy (CLM) over previous ones are the speed-up of the image acquisition by over one order of magnitude, and the capability of monitoring the sample through conventional (i.e., intensity-based) diffraction-limited microscopy.

The comparison reported in Table 1 clarifies the expected theoretical improvements offered by CLM, in terms of both resolution and DOF^44^, with respect to both standard microscopy and conventional light-field microscopy^46^. The first column highlights the diffraction-limited imaging capability that CLM shares with conventional microscopy, as opposed to the sacrificed image resolution of conventional light-field imaging. The factor $N_u$ that quantifies the resolution loss of conventional light-field imaging is defined by the number of resolution cells, per side, dedicated to directional information, and is proportional to the DOF improvement. The second and third columns of the table report the DOF of the three methods, respectively, for object details at the resolution limit and object details of arbitrary size. In light-field imaging, the latter represents the refocusing range, while the former determines the axial resolution in the focused plane. In conventional light-field microscopy, increasing the refocusing range (i.e., choosing large values of $N_u$) entails a proportional loss of transverse resolution, and an even more detrimental loss of axial resolution (proportional to $N_u^2$); this generally limits $N_u$ to values smaller than 10. Furthermore, for object details larger than the resolution limit, both the DOF of standard microscopy and the refocusing range of conventional light-field microscopy scale linearly with the size of the object details; this is due to the “circle of confusion” generated by the finite numerical aperture of the imaging system. In CLM, instead, the refocusing range scales quadratically with the size of object details, and is only limited by diffraction at the object (see Refs. ^33,37,44^ for a detailed discussion; specifically, the DOF extension for CLM derives from Eq. (23) of Ref. ^44^). These are key features in ensuring the unique refocusing advantage of CLM. The fourth column represents the axial resolution of the three techniques, as defined by the circle of confusion (see “Materials and methods” section for details). The ratio between the third and the fourth columns can be regarded as the number of independent axial planes that each technique is capable of providing: The axial resolution is the same for all three imaging methods, but the scaling of the DOF with the square of the object resolution *a*, in CLM, implies a linear scaling of the number of independent axial planes with *a*; on the contrary, in standard light-field, the number of independent axial planes is fixed by $N_u$, which is generally significantly smaller than $3.3 \mathrm {NA}_0 a/\lambda$. The last column of Table 1 also indicates that the refocusing range of both light-field microscopes is strictly related with the viewpoint multiplicity, defined as the number of available viewpoints, per side, on the three-dimensional sample: In all the considered imaging modalities, the viewpoint multiplicity is proportional to the aforementioned number of independent axial planes that can be refocused, given the size of the details of interest. In sharp contrast with conventional light-field microscopy, the viewpoint multiplicity of CLM can be even one order of magnitude larger than in conventional light-field imaging, without the diffraction-limited resolution to be affected; this is especially true for imaging systems with a large numerical aperture and for refocusing far away from the focused plane (i.e., for large values of object details *a*). Most important, the DOF extension capability of CLM is independent on the numerical aperture of the imaging systems (see “Materials and methods” for details); a large numerical aperture can thus be chosen for maximizing the volumetric resolution without affecting the DOF. This is very different from conventional light-field microscopy, where the numerical aperture and $N_u$ need to be properly chosen to achieve an acceptable compromise of resolution and DOF.

**Table 1:** Comparison of resolution and DOF limits of three microscopy techniques: standard microscopy (with no directional resolution), standard light-field microscopy, and CLM.

Microscopy method	Resolution limit	DOF at resolution limit	DOF extension at object detail size *a*	Axial resolution at object detail size *a*	Viewpoint multiplicity
Standard	$\Delta x_0$	$\displaystyle \frac{\Delta x_0}{\mathrm {NA}_0}$	$\displaystyle \frac{a}{\mathrm {NA}_0}$	$\displaystyle \frac{a}{\mathrm {NA}_0}$	1
Light-field	$N_u\Delta x_0$	$\displaystyle \frac{N_u^2\Delta x_0}{\mathrm {NA}_0}$	$N_u\displaystyle \frac{a}{\mathrm {NA}_0}$	$\displaystyle \frac{a}{\mathrm {NA}_0}$	$N_u$
CLM	$\Delta x_0$	$\displaystyle \frac{\Delta x_0}{\mathrm {NA}_0}$	$\displaystyle 3.3 \,\frac{a^2}{\lambda }$	$\displaystyle \frac{a}{\mathrm {NA}_0}$	$\displaystyle \frac{a}{\Delta x_0}$

Here, $\lambda$ is the light wavelength and $\Delta x_0=0.61\,\lambda /\mathrm {NA}_0$ the diffraction-limited resolution cell, with $\mathrm {NA}_0$ the numerical aperture of the microscope. $N_u$ is the number of directional resolution cells per side in standard light-field imaging, and *a* is the size of the smallest details within the sample. The first and second columns represent the resolution limit in the focal plane and the maximum DOF achievable for objects with details at the resolution limit, respectively. The third column indicates the maximum DOF achievable for objects with detail size $a>\Delta x_0$ larger than the resolution limit. The fourth column represents the axial resolution for objects of detail size *a*, which, as shown in the “Materials and methods” section, does not depend on the specific technique. The last column reports the number of viewpoints per direction. Properties of the light-field microscope are derived by the general features of light-field imaging devices (see Ref.^46^ for a detailed discussion), while the resolution and DOF limits in CLM are derived from the theoretical analysis reported in Ref. ^44^, as well as in the “Materials and methods” section. Notice that all the quantities are evaluated in the limit of small displacements with respect to the objective focal plane.

The paper is organized as follows. In the “Results” section, we outline the theoretical basis of the method and show the experimental results. In the “Discussion” section, we discuss the results, their impact on state of the art, and the possibilities of further improvement. In the “Materials and methods” section, we provide a detailed description of the experimental setup and on the methods to extract relevant information from correlation measurements.

## Results

### Concept

**Figure 1:**
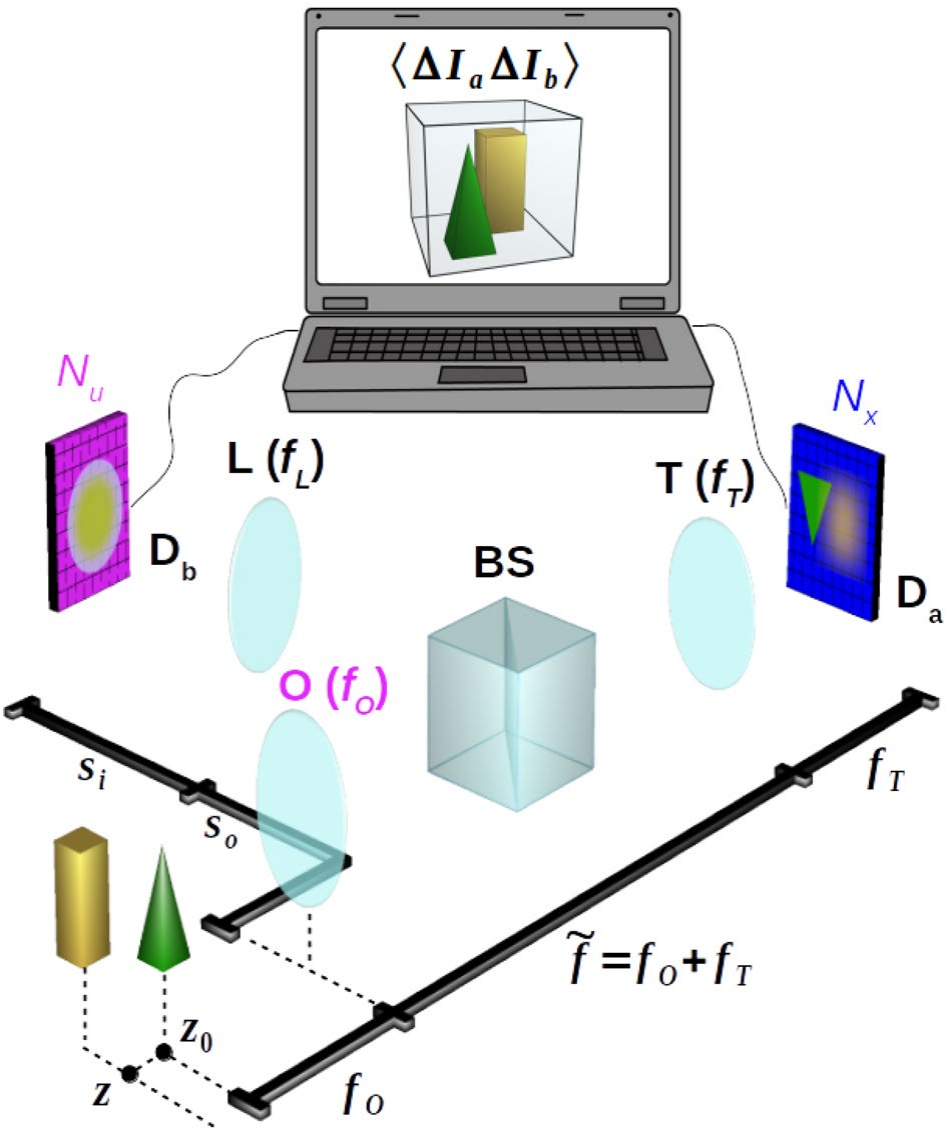
Schematic representation of light-field microscopy with correlated beams. Light from the sample (green pyramid and yellow parallelepiped) is split in two optical paths by a beam splitter (BS), placed after the objective lens (O). The microscope can image only the part of the sample which is at focus (green), while the part outside the DOF is blurred (yellow). Along the transmitted path, the tube lens (T) focuses on detector $\mathrm {D}_a$ (blue) the 3D sample, with a DOF defined by the numerical aperture of the objective. Along the reflected path of the BS, the objective lens is imaged on detector $\mathrm {D}_b$ (magenta) by means of an additional lens (L). The intensity patterns registered by the two array detectors, in a collection of *N* frames, are processed by a computer to reconstruct the correlation function (Eq. (1)) encoding three-dimensional plenoptic information on the sample. All experimental results presented throughout the paper have been obtained by employing this setup.

The correlation light-field microscope, schematically represented in Fig. 1, is based on a conventional microscope made of an objective lens (O) and a tube lens (T) to reproduce the image of the sample on a high resolution sensor array (detector $\mathrm {D}_a$); this microscope can properly reconstruct only the slice of the three-dimensional object falling within its DOF. The capability of CLM to refocus out-of-focus parts of the three-dimensional sample comes from its ability to also gain directional information about light coming from the sample. In our architecture, this is done by means of the beam splitter (BS) that reflects a fraction of light emerging from the objective lens toward an additional lens (L), which images the objective lens on a second high resolution sensor array (detector $\mathrm {D}_b$). Further details on the experimental setup are reported in the “Materials and methods” section.

The three-dimensional sample is a chaotic light emitter or, alternatively, a diffusive, transmissive, or reflective sample illuminated by an external chaotic light source. The chaotic nature of light enables light-field imaging thanks to the rich information encoded in *correlations between intensity fluctuations*. In fact, the intensity retrieved by pixels simultaneously illuminated on the two disjoint detectors $\mathrm {D}_a$ and $\mathrm {D}_b$ is employed in CLM to evaluate the correlation function

1 $$\begin{aligned} \Gamma (\varvec{\rho }_a,\varvec{\rho }_b) = \langle \Delta I_a (\varvec{\rho }_a) \Delta I_b (\varvec{\rho }_b) \rangle , \end{aligned}$$

where $\langle \dots \rangle$ is the average over the source statistics, $I_{a}(\varvec{\rho }_{a})$ and $I_{b}(\varvec{\rho }_{b})$ are the intensities at the transverse positions $\varvec{\rho }_a$ and $\varvec{\rho }_b$ on detectors $\mathrm {D}_a$ and $\mathrm {D}_b$, within the same frame, respectively, and $\Delta I_{j} (\varvec{\rho }_{j}) = I_{j}(\varvec{\rho }_{j}) - \langle I_{j} (\varvec{\rho }_{j}) \rangle$, $j=a,b$. The statistical reconstruction of the correlation function requires, under the hypotheses of a stationary and ergodic source, to collect a set of *N* independent frames. In the best-case scenario, the exposure time of each frame is matched with the coherence time of the source.

**Figure 2:**
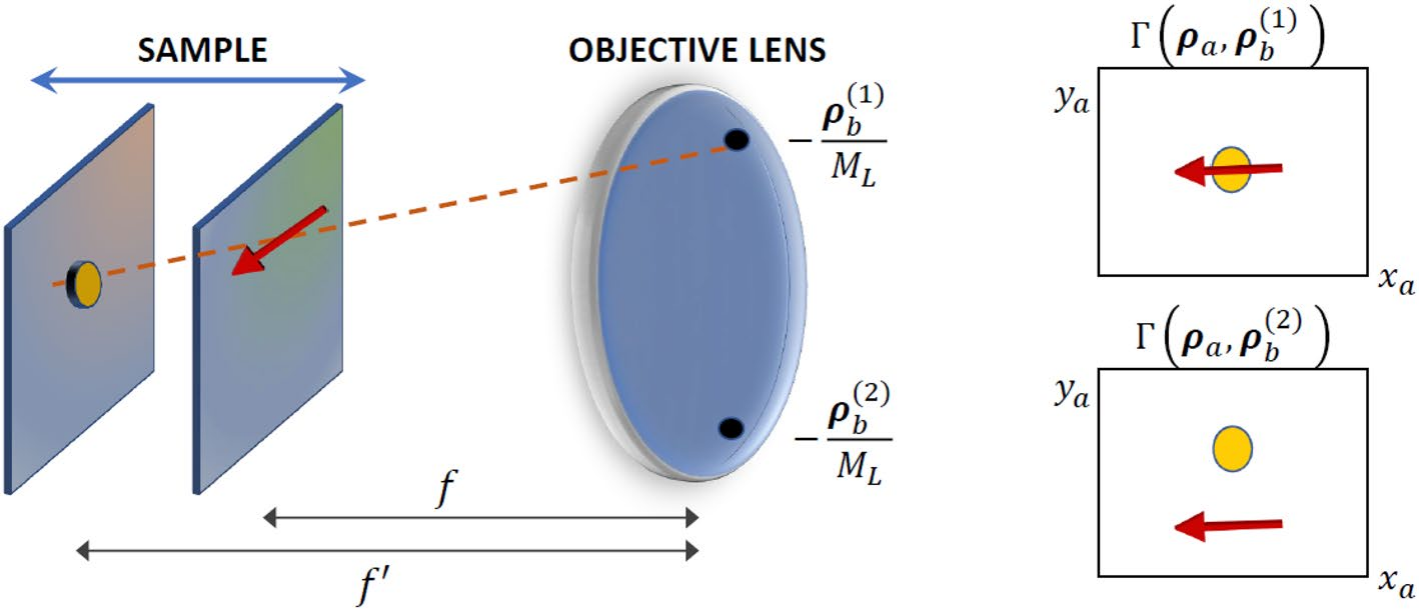
Schematic representation of the different points of view on the 3D sample that the correlation function $\Gamma (\varvec{\rho }_a,\varvec{\rho }_b)$ incorporate (Eq. (2)); here, $\varvec{\rho }_i=(x_i, y_i)$, $i=a,b$. A thick sample is represented through a (yellow) circle and a (red) arrow placed at distances *f* and $f^{\prime}$, respectively, from the objective lens. These two details can appear superposed (upper panel on the right) or well separated (lower panel on the right) depending on the specific transverse coordinate $\varvec{\rho }_b$ chosen on detector $\mathrm {D}_b$, that corresponds to a point $-\varvec{\rho }_b/M_L$ on the objective lens.

The light-field imaging capability of CLM explicitly emerges when considering the geometrical optics limit of the above correlation function, which reads^44,45^

2 $$\begin{aligned} \Gamma (\varvec{\rho }_a,\varvec{\rho }_b) \sim F^2 \! \left( - \frac{f}{f_T} \varvec{\rho }_a - \left( 1 - \frac{f}{f_O} \right) \frac{\varvec{\rho }_b}{M_L} \right) P^2 \left( - \frac{\varvec{\rho }_b}{M_L} \right) \end{aligned}$$

where $F(\varvec{\rho }_s)$ is the intensity profile of light from the sample, $P(\varvec{\rho }_O)$ is the intensity transmission function of the objective, *f* is the distance from the objective of the generic plane within the three-dimensional object, $f_T$ is the focal length of the tube lens, and $M_L$ is the magnification of the image of the objective lens retrieved by $\mathrm {D}_b$. When the plane of interest is on focus (i.e., $f=f_O$, with $f_O$ the focal length of the objective), the correlation simply gives a focused image identical to the one retrieved by detector $\mathrm {D}_a$. However, as shown in Fig. 2, points of the three-dimensional samples that are out-of-focus (i.e., they lie in planes at a distance $f \ne f_O$ from the objective) are seen as shifted, and their displacement depends on the specific pixel $\varvec{\rho }_b$ chosen on sensor $\mathrm {D}_b$, corresponding to the point $\varvec{\rho }_O=-\varvec{\rho }_b/M_{L}$ on the objective. In other words, for three-dimensional samples that are thicker than the natural DOF of the microscope, different values of $\varvec{\rho }_b$ correspond to different choices of the point of view on the sample: The correlation function in Eq. (1) has the form of a four-dimensional array, characterized by both detector coordinates $(x_a, y_a, x_b, y_b)$, encoding all the spatial and angular information needed for refocusing and multi-perspective image. By fixing the coordinates $(x_b,y_b)$ of the 4D array, one makes a “slice” of the correlation function, which corresponds to selecting an image of the sample from a chosen viewpoint on the objective lens. This property enables to detect the position of sample details, in three dimensions, and to highlight hidden parts of the sample. The refocused image of a sample plane placed at an arbitrary distance *f* from the objective can be obtained by properly stacking and summing such different perspectives^44,45^:

3 $$\begin{aligned} \Sigma _{\mathrm {ref}} (\varvec{\rho }_a) = \int \mathrm {d}^2\varvec{\rho }_b \Gamma \left( \frac{f_O}{f} \varvec{\rho }_a + \left( 1- \frac{f_O}{f} \right) \frac{M}{M_L} \varvec{\rho }_b , \varvec{\rho }_b \right) \sim F^2 \! \left( - \frac{\varvec{\rho }_a}{M} \right) \end{aligned}$$

with $M=f_T/f_O$ being the natural microscope magnification. The refocusing procedure increases significantly the signal-to-noise ratio (SNR) with respect to the one characterizing the single perspective associated with $\varvec{\rho }_b$. Notice that the technique can be generalized to the case of samples with axially varying diffraction index, upon replacing physical distances with optical distances. Moreover, reconstruction of the correlation function was proved to be robust against the presence of turbulence and scattering surrounding the sample, within a limit distance defined by the emitted light wavelength and transverse coherence^44^.

Before moving on to the experimental demonstration of CLM, let us spend a few words on the advantages offered by the capability of CLM to collect a large number of viewpoints, as enabled by the large objective plane on which perspectives can be selected, and by the consequent wide angle from which the sample can be observed. First, refocusing shares with 3D imaging the close connection between maximum observation angles and achievable DOF. Second, the higher the number of points of view that are superimposed to obtain the refocused image (see the integration over $\rho _b$ in Eq. (3)), the more effective will be both the enhancement of features on the plane of interest and the suppression of contributions from neighboring planes, as well known in 3D imaging. Moreover, superimposing a large number of perspectives to form the final image is advantageous in terms of noise reduction. Indeed, when points of view with statistically independent noise are summed, the process results in a point-wise increase of the signal-to-noise ratio that is proportional to the square root of the number of contributions.

### Volumetric imaging by CLM

The refocusing and depth mapping capability of CLM has preliminarly been tested with a simple and controllable three-dimensional object, made of two planar resolution targets (named $3\mathrm {D}_1$ and $3\mathrm {D}_2$) placed at two different distances from the objective lens, well outside its natural DOF. Data have been acquired by focusing the correlation light-field microscope onto a plane that did not contain neither one of the two test targets. Correlation measurements, combined with Eq. (3), has been employed to refocus the two test targets, separately, starting from this dataset. The illuminated parts of both targets contain triple slits with center-to-center distance $d = 49.6\,\upmu \mathrm {m}$ and slit width $a=d/2$; the overall linear field of view (FOV) is $0.54\,\mathrm {mm}$. The test targets are placed at a distance of $2.5\, \mathrm {mm}$ from each other (i.e. $f_{\text {3D}_1} - f_O = -1250\,\mathrm {\upmu m}$ and $f_{\text {3D}_2}-f_O = 1250\,\mathrm {\upmu m}$, where $f_O$ is the focal length of the objective lens), which is 6 times larger than the natural DOF of the microscope at the given size of the sample details (i.e., the circle of confusion, see Table 1). The reported results have been obtained by evaluating the correlation function over $N=5\times 10^3$ acquired frames; we report in the Supplementary Information further details on SNR variation with the number of collected frames.

**Figure 3:**
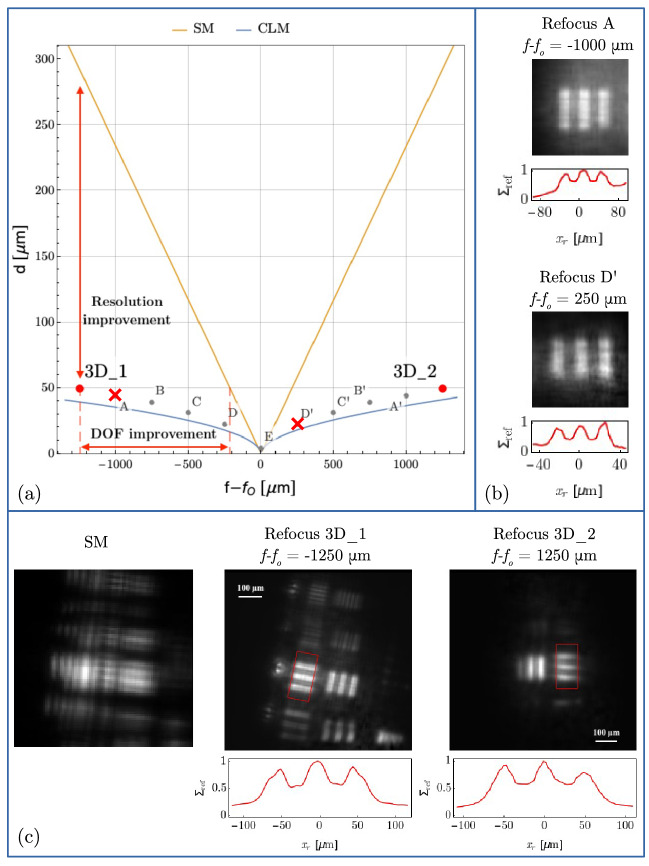
Panel (**a**) compares the resolution versus DOF compromise in a standard microscope (SM) and in CLM, with a numerical aperture $\mathrm {NA}_0=0.23$ and illumination wavelength $\lambda =532\ \mathrm {nm}$. The curves represent the limit at which a double-slit mask with center-to-center distance *d* (reported on the vertical axis and equal to twice the slit width) can be discriminated with a visibility of 10%, as a function of the longitudinal distance of the mask from the objective focal plane ($f-f_O$). The curves are obtained assuming the same illumination wavelength and numerical aperture for the two microscopes. Points $3D_1$ and $3D_2$ indicate the parameters of a three-dimensional sample made of two planar resolution targets (triple slits with slit distance $d=49.6\,\upmu \mathrm {m}$) placed at a distance $f_1-f_O = -1250\,\upmu \mathrm {m}$ and $f_2-f_O = 1250\,\upmu \mathrm {m}$, respectively, from the objective. Points A to E, and A’ to D’ correspond to further experimental data demonstrating the expected maximum achievable DOF of CLM at different resolutions, as the object (a resolution test target) is moved away from the plane at focus (A, A’: $d= 44.2\,\upmu \mathrm {m}$ at $f-f_O= \pm 1000\,\upmu \mathrm {m}$; B, B’: $d= 39.4\,\upmu \mathrm {m}$ at $f-f_O= \pm 750\,\upmu \mathrm {m}$; C, C’: $d= 31.3\,\upmu \mathrm {m}$ at $f-f_O= \pm 500\,\upmu \mathrm {m}$; D, D’: $d= 22.1\,\upmu \mathrm {m}$ at $f-f_O= \pm 250\,\upmu \mathrm {m}$, E: $d= 4\,\upmu \mathrm {m}$ at $f-f_O=0\,\upmu \mathrm {m}$). The refocused images of the triple slits corresponding to points A and D’ are shown in panel (**b**), while the other cases are reported in the Supplementary Information. The line plots below the refocused images show the image intensities, averaged along the vertical directions and normalized to their maxima. Panel (**c**) reports the images of the three-dimensional test sample described above, corresponding to points $3D_1$ and $3D_2$ in panel (**a**): image acquired by the standard microscope (left), CLM refocusing on the plane of the closest and farthest target, $3\mathrm {D}_1$ (center) and $3\mathrm {D}_2$ (right), respectively. The line plots below the refocused images show the intensities within the regions boxed in red, averaged along the longitudinal slit direction and normalized to their maxima.

The improvement of CLM over standard microscopy can be observed in Fig. 3a, where we report the resolution-versus-DOF compromise in the two microscopes. In particular, the curves indicate the resolution limits of CLM and a standard microscope (SM) with the same numerical aperture, as a function of the distance from the objective focal plane. The resolution limit is defined in both cases as the value *d* of the center-to-center distance between two slits of width $a=d/2$, such that their images can be discriminated at 10% visibility; such a definition generalizes the Rayleigh criterion for resolution to out-of-focus images. For a fixed slit separation *d* (vertical axis), one can identify the longitudinal range $f-f_O$ (horizontal axis) where the images of the two slits can be discriminated, based on our theoretical results. Points labeled from A to E and from A’ to D’ in Fig. 3a were experimentally investigated to demonstrate the agreement of the experimental results with the theoretical prediction of the resolution and DOF limits. We explored the range between $-1$ mm and $+1$ mm along the optical axis, in steps of $250\ \upmu$m, by employing different triple-slit masks of a planar resolution test target, characterized by center-to-center distances ranging from $44\, \upmu \mathrm {m}$ (A and A’) to $4\, \upmu \mathrm {m}$ (E). In particular, point E is close to the diffraction limit of a standard microscope and shows that CLM is capable of the same resolution at focus. The experimental validation of the CLM refocusing capability for the cases A and D’ is reported in Fig. 3b; the refocused images obtained in all the other cases are reported for completeness in the Supplementary Information. The red points in Fig. 3a identify the parameters of the resolution targets $3\mathrm {D}_1$ and $3\mathrm {D}_2$ that compose our three-dimensional test object. The successful experimental refocusing of both targets, reported in Fig. 3c, demonstrates that CLM enables achieving a 6 times larger DOF than standard microscopy, at the given resolution, or, alternatively, a 6 time better resolution, at the given DOF. The leftmost panel reports for comparison the standard microscope image of the three-dimensional test object, in which both target planes are clearly out of focus and none of the triple-slit groups can be recognized. Remarkably, the sets of triple slits placed at $f_{\text {3D}_2}-f_O=1250\,\upmu \mathrm {m}$ (i.e., the object placed farthest from the objective) are still perfectly resolved. This means that the resolution achieved on farther planes is not heavily influenced by the presence of other details placed along the optical path, despite the substantial spatial filtering that they perform.

The results in Fig. 3c also demonstrate that CLM improves by over one order of magnitude the acquisition speed with respect to previous correlation-based light-field imaging protocols^37,38^, where $5\times 10^4$ frames (to be compared with the current $5\times 10^3$) and additional low-pass Gaussian filtering were employed in post-processing to achieve a comparable SNR. This improvement directly comes from the elimination of ghost imaging from the CLM architecture, and its replacement by conventional imaging at both sensor arrays. Actually, correlation between direct images has been shown to enable a significant improvement of the SNR with respect to ghost imaging^38^.

**Figure 4:**
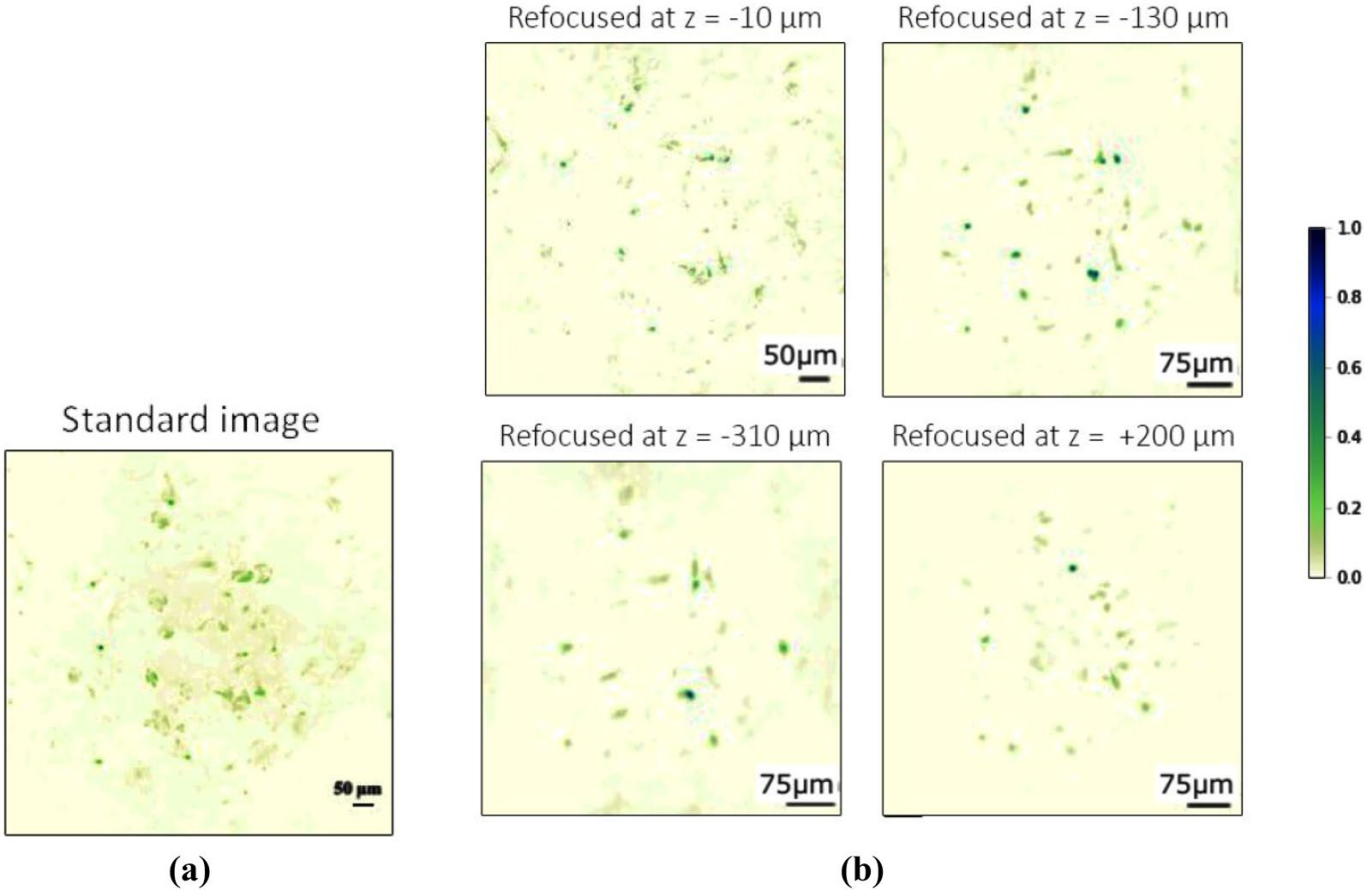
First-order image of a thick biomedical phantom (**a**) acquired at $f-f_O=0$ $\upmu \mathrm {m}$, and refocused CLM images of four distinct planes inside the same sample (**b**); the specific value of $z=f-f_O$ is reported on top of each image. All images have been obtained from the same data, using $N=5000$ frames. The technique used to optimize the correlation function in this case is described in the “Materials and methods” section.

After the three-dimensional target, we tested the effectiveness of CLM on a thick phantom reproducing features of interest in biomedical applications; the sample is made of birefringent starch granules, suspended at random positions in a transparent non-birefringent gel. The focused plane inside the sample was arbitrarily chosen at approximately half of its thickness. In Fig. 4a, we show the standard image of the focused plane, while Fig. 4b reports the images of four different planes refocused by CLM, located at an optical distance from the focused plane of $-10\,\upmu \mathrm {m}$, $-130\,\upmu \mathrm {m}$, $-310\,\upmu \mathrm {m}$, and $+200\,\upmu \mathrm {m}$, respectively. It is evident that some aggregates appear focused in only one of the four images, which provide a tool to identify their longitudinal optical distance from the focal plane. The volumetric resolution of CLM enabled us to refocus 54 planes over a $1\,\mathrm {mm}$ thick volume, with a transverse resolution smaller than $20\,\upmu \mathrm {m}$ and a longitudinal resolution smaller than $90\,\upmu \mathrm {m}$, within a FOV of about $1\,\mathrm {mm}^2$ (see video in the Supplementary Information).

**Figure 5:**
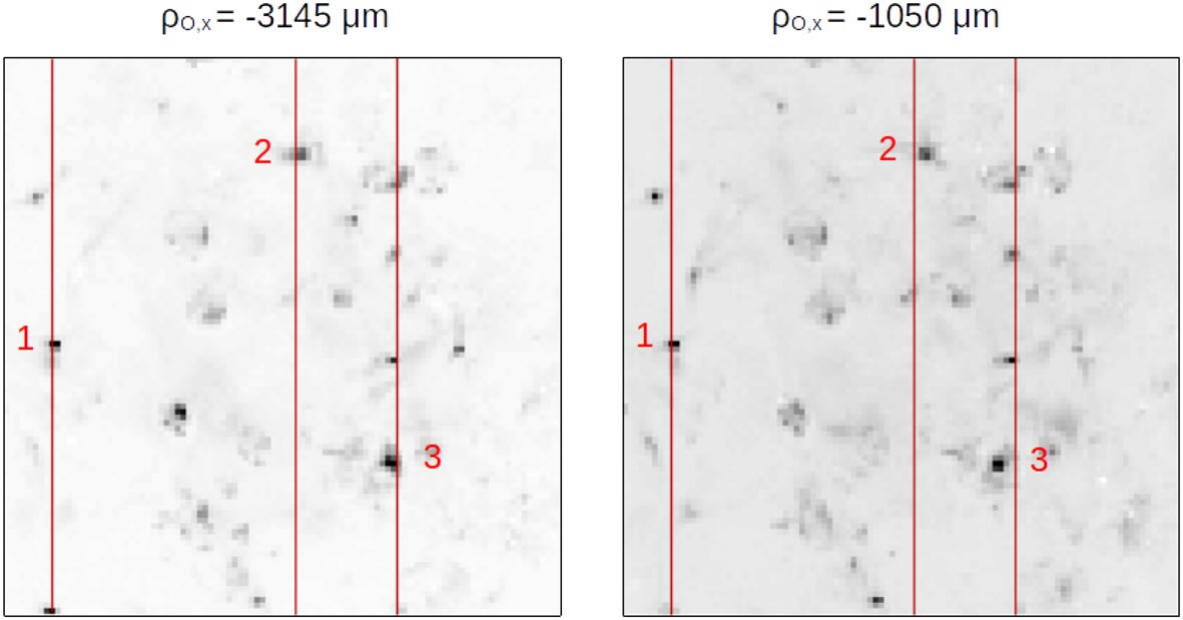
Change of perspective within the $1\, \mathrm {mm}^3$ sample of starch grains suspended in gel, as obtained through CLM. The change of perspective is implemented by correlating the intensity registered by each pixel of the spatial sensor ($D_a$) with the intensity registered by a small portion ($10 \times 10$ pixels) of the angular sensors ($D_b$), centered on the point $\varvec{\rho }_b = (-M\rho _{O,x},0)$, corresponding to the horizontal coordinate $\rho _{O,x}$ on the objective lens [i.e., by varying the second argument in Eq. (2)]. Two different viewpoints of the sample are shown, selected on the horizontal diameter of the objective lens. The image on the left is the perspective from a point placed 3145 $\upmu$m to the left of the center of the lens, corresponding to an angle of $-5^{\circ }$ from the optical axis (OA). The one on the right is the viewpoint at $-1050\,\upmu$m ($-2^{\circ }$ with respect to OA). Three different features are highlighted. The starch granule labeled by 1 does not shift with a change of viewpoint, meaning it is placed on the focal plane of the objective. The granule labeled with 2 shifts to the right as the viewpoint shifts to the right, meaning it is placed (out of focus) on a plane between the lens and its focal plane. The granule labeled with 3, on the other hand, suffers a shift in the opposite direction, meaning its longitudinal distance from the lens is larger than the focal length. As one would expect from a viewpoint change along the horizontal, none of the visible granules change their position along the vertical axis.

Interestingly, in the current CLM architecture, the SNR is high enough for images from different viewpoints to be effectively observable (hence, available for further data analysis, such as three-dimensional reconstruction). In Fig. 5, we report the change of perspective obtained by CLM when moving the “viewpoint” on the objective lens plane, along the horizontal direction: While the position of details at focus does not change with the particular perspective, out-of-focus starch granules shift along the horizontal direction as the point of view is changed. Through a single correlation image, we have acquired 130,000 images of the sample from different viewpoints, distributed over the area of the objective lens ($\sim 1\,\mathrm {cm}^2$), each one characterized by a diffraction-limited spatial resolution of $40\,\upmu \mathrm {m}$. Such a high number of statistically independent perspectives has allowed us to produce viewpoints images in which the details of the object can be clearly distinguished, a feature which is particularly relevant in view of implementing 3D reconstruction algorithms based on viewpoint multiplicity.

## Discussion

The refocusing capability of CLM has brought to a 6 times larger DOF than in a conventional microscope having the same numerical aperture, and the same (diffraction-limited) resolution in the focused plane. These results are in excellent agreement with the expected refocusing range of CLM^44^ at the given resolution ($d=50\,\upmu \mathrm {m}$), thus showing the reliability of the proposed CLM architecture. The volumetric resolution achieved by CLM to image a complex thick sample ($1 \times 1 \times 1 \,\mathrm {mm}^3$) is a further very interesting result, considering the scanning-free nature of CLM. In the employed CLM setup, we obtained 54 independent axial sections of the biological phantom. Considering how the axial and transverse resolution change along the optical axis, this corresponds to a total $7.21\times 10^6$ voxels within the volume of interest. Notice that the device considered in this work is meant as a proof-of-principle demonstrator, and its parameters are not optimized. However, the properties reported in Table 1 provide a guideline to scale the setup towards smaller resolutions in view of real applications. All these results are unattainable by standard light-field microscopy, due to its hyperbolic tradeoff between spatial resolution and multi-perspective views (hence, maximum achievable DOF).

CLM shares with traditional light-field microscopy the capability of performing three-dimensional imaging without moving the sample nor any part of the optical apparatus; resolution depends on the distance from the focal plane, as shown in Fig. 3, but is uniform in the transverse directions, as far as light propagation can be considered paraxial^44^. These features can be compared to the properties of a consolidated technique such as confocal microscopy, which requires both longitudinal and transverse scanning to perform imaging with uniform volumetric resolution, and of a much less time-consuming technique such as light-sheet microscopy with non-diffracting beams, which requires only longitudinal scanning, trading this interesting feature with inhomogeneous illumination and resolution in the transverse direction. The main drawback of CLM lies in its operational definition: while in incoherent first-order imaging the SNR can be increased by just exposing the sensor for a time much larger than the source coherence time, a reliable reconstruction of the correlation function (1) requires to collect a large number of distinct frames, whose duration should be preferably matched with the source coherence time.

Increasing the acquisition speed of CLM is the principal challenge that needs to be addressed to guarantee its competitiveness with state-of-the art light-field microscopes^25^. Such speed-up is in fact of paramount importance both for avoiding radiation damage of biomedical samples, for *in vivo* imaging, and for studying dynamic processes. The large SNR of CLM with respect to the original CPI scheme represents a first significant step in this direction, as it enabled to increase the acquisition speed by one order of magnitude, still guaranteeing an even higher SNR (see Ref. ^33^). Similar to the approach for noise mitigation implemented here (Figs. 4, 5), and outlined in the “Materials and methods” section, a further step toward acquisition speed-up is compressive sensing and deep learning techniques, as increasingly applied to imaging tasks^47–50^. From the hardware viewpoint, the acquisition speed of our microscope has ample room for improving, both by investigating possible optimizations in our current acquisition routine and by employing cameras with better time performance. The most immediate way to start boosting the time performance of the current CLM, for example, is to employ the camera (see “Materials and methods” section) in rolling-shutter mode, rather than global shutter, which we have been using to guarantee that the retrieved intensity patterns $I_a$ and $I_b$ (which are then correlated pixel by pixel) are simultaneous statistical sampling of the chaotic source. This condition is consistent with the theoretical model (i.e., Eq. (1)), but it is certainly interesting to search for a regime in which moving slightly away from the theory introduces small enough artifacts to justify the gain in speed. With our camera, this could mean even doubling the frame rate, reducing the current acquisition time to about 20 seconds (from the present 43). Also the chaotic source can be significantly improved by replacing the ground-glass disk now in use (see the “Materials and methods” section) with a digital micromirror device (DMD), which adds versatility and available statistics, while significantly decreasing the source coherence time due to its typical frame rate of about 30 kHz. Also, since the DMD patterns are completely user-controllable, their features can be customized to achieve the desired SNR with the lowest number of frames possible, even experimenting with structured illumination. In this scenario, the acquisition speed will essentially be limited by the maximum frame rate of the sensor and, eventually, by the data transferring speed. This issue can be addressed by replacing our current sCMOS with faster cameras, capable of reaching 6.6 kfps at full resolution^51^, or with ultra-fast high-resolution SPAD arrays, enabling acquisition rates as high as $10^5$ binary frames per second, in a $512 \times 512$ array^52,53^. When choosing alternative cameras, speed should not be favored at the expenses of readout noise, dynamic range, detection efficiency, or minimum exposure time, all which are relevant parameters in correlation-based imaging. In this respect, SPAD arrays are of particular interest due to their much shorter minimum exposure time, ranging from a few hundreds of ps to 10 ns^52–55^, although their binary nature may pose challenges. The minimum exposure time of the camera also regulates the possibility of extending CLM to uncontrolled thermal sources, including the fluorescence samples at the core of the related microscopy technique. CLM and fluorescence microscopy are certainly compatible due to the chaotic nature of fluorescent light, but an experimental challenge needs to be addressed in such a context: matching the low coherence time of fluorescent light with the minimum exposure time of the sensor. An analogous problem was successfully faced by Hanbury-Brown and Twiss in the Narrabri stellar interferometer^56^ and in more recent correlation imaging experiments performed with sunlight^57,58^. In the context of CLM, we shall address this challenge in future works.

We finally remark that the need for many short exposure times to create a single image is common to other established microscopy techniques, such as STORM^59^. This result is encouraging in view of avoiding photo-bleaching and photo-damage, also considering that the SNR requirements on each single frame of CLM are much weaker than in STORM, where the signal should be clear enough to allow centroid estimation. Exposure-related problem could also be reduced by modulating illumination in time, with a frequency matched with the acquisition frame rate.

## Materials and methods

### Experimental setup

**Figure 6:**
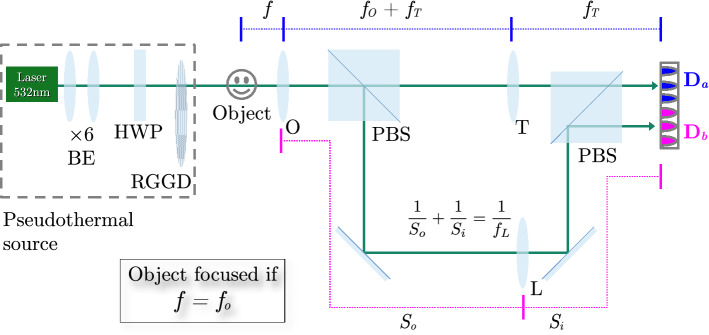
Experimental setup for CLM. Light produced by a chaotic source, made of a laser, a beam expander (BE), a half-wave plate (HWP) and a rotating ground-glass disk (RGGD), illuminates the object, is collected by the objective lens (O), and reaches the first polarizing beam splitter (PBS). The transmitted beam passes trough the tube lens (T) and the second PBS, and finally impinges on the detector $\mathrm {D}_a$, which is the (blue) half of the sensor of the CMOS camera. The focused image is characterized by magnification $M = f_T/f_O = 4.2$. The reflected beam passes through the additional lens L and then is reflected by a second PBS toward detector $\mathrm {D}_b$, which is the disjoint (magenta) half of the sensor on the same sCMOS camera. The magnification of the objective lens O on $\mathrm {D}_b$ is $M_L=0.31$. All the experimental results shown in the article are obtained in this setup.

The experimental setup employed to demonstrate CLM is shown in Fig. 6. The controllable chaotic light source is a single-mode laser with wavelength $\lambda =532\,\mathrm {nm}$ (CNI MLL-III-532-300 mW) illuminating a rotating ground glass disk (GGD), with diffusion angle $\theta _d \simeq 14^{\circ }$, whose speed defines the source coherence time ($\approx 90 \upmu$s). The laser spot size on the disk is enlarged to a diameter of 8 mm by a $6 \times$ beam expander, and the sample is placed at a distance of $10\,\mathrm {mm}$ after the GGD; the effective numerical aperture of our systems is thus $\mathrm {NA} = 0.23$ which defines our expected diffraction-limited resolution $\delta = 1.6\,\upmu \mathrm {m}$. Light transmitted by the object propagates toward the objective lens O, with focal length $f_O = 30\,\mathrm {mm}$, and reaches the first polarizing beam splitter (PBS) where it is divided in two beams. The transmitted beam reaches the tube lens T, with focal length $f_T = 125\,\mathrm {mm}$, and then impinges on the part of the sensor identified with $\mathrm {D}_a$. The distance between the objective lens O and the tube lens T is equal to the sum of the focal lengths of the two lenses, $f_O + f_T$, and the distance between T and $\mathrm {D}_a$ coincides with $f_T$. The focused image plane thus lies at a distance $f_O$ from the objective lens. The beam reflected off the PBS illuminates lens L, with focal length $f_L = 150\,\mathrm {mm}$, then impinges on the part of the sensor identified with $\mathrm {D}_b$, after being reflected by the second PBS. The distance $S_O$ between the objective lens O and the lens L, and the distance $S_I$ between L and $\mathrm {D}_b$ are conjugated and the front aperture of the objective is imaged on $\mathrm {D}_b$. The measured magnification of such image is $M_L = 0.31$. Two disjoint halves of the same camera (Andor Zyla 5.5 sCMOS) are employed to simulate the two sensors $\mathrm {D}_a$ and $\mathrm {D}_b$, in order to guarantee synchronization. To fully exploit the dynamic range of the camera and maximize the SNR, we balance the intensities of the beams on the two halves of the sCMOS camera by means of a half-wave plate placed before in the laser beam, before the GGD. The camera sensor is characterized by $2560\times 2160$ pixels of size $\delta _{\mathrm {p}} = 6.5\,\upmu \mathrm {m}$ and can work at up to 50 fps in full-frame mode (in global shutter mode, 100 fps with rolling shutter). Since the resolution $\delta = 1.6\,\upmu \mathrm {m}$ on the object corresponds to a magnified resolution cell $M\delta = 6.7\,\upmu \mathrm {m}$ on the sensor, data reported in Figs. 4, 5 were generally acquired with a $2\times 2$ hardware binning; no binning was applied when acquiring data corresponding to points C, D, their primed counterparts, and E, in Fig. 3a. The test targets employed to acquire data reported in Fig. 3 are Thorlabs R3L3S1N and R1DS1N. The exposure time was set at $\tau = 92.3\,\upmu \mathrm {s}$ to match the coherence time fo the source, and the acquisition rate of the camera to $R = 120\,\mathrm {Hz}$, the maximum speed possible at our FOV in global shutter mode.

### Noise mitigation in the CLM correlation function

All the refocused images reported in the article are obtained by applying the refocusing formula in Eq. (3) to the experimental four-dimensional correlation function. We also applied a correction to eliminate edge-effects due to the size of the detectors, as described in Ref. ^45^. The problem of the noisy background occurring in the refocused images of the biomedical phantom (Figs. 4, 5) was tackled by pre-processing the correlation function. The statistical noise, which is quantified by the variance of the quantity measured in Eq. (1), can be reduced by optimizing the correlation function with the introduction of an additional term; this approach is consistent with so called *differential ghost imaging*^60^, where each pixel of $\mathrm {D}_b$ is considered as a bucket detector. The mentioned correction consists in subtracting from the correlation function a spurious self-correlation between intensity fluctuations on each single pixel of the spatial detector $\mathrm {D}_a$ and intensity fluctuations on the whole detector, thus obtaining the modified correlation

4 $$\begin{aligned} \widetilde{\Gamma }(\varvec{\rho }_a,\varvec{\rho }_b) = \Gamma (\varvec{\rho }_a,\varvec{\rho }_b)- K\left\langle \Delta I_a (\varvec{\rho }_a) \Delta I_a^{\mathrm {TOT}} \right\rangle = \left\langle \Delta I_a (\varvec{\rho }_a) \left( \Delta I_b (\varvec{\rho }_b) - K \Delta I_a^{\mathrm {TOT}} \right) \right\rangle . \end{aligned}$$

with $I_a^{\mathrm {TOT}}$ the total intensity impinging on the detector $\mathrm {D}_a$. The free parameter *K* can be fixed by the condition of minimizing the variance

5 $$\begin{aligned} \mathcal {F}(\varvec{\rho }_a,\varvec{\rho }_b) = \left\langle \left[ \Delta I_a (\varvec{\rho }_a) \left( \Delta I_b (\varvec{\rho }_b) - K \Delta I_a^{\mathrm {TOT}} \right) \right] ^2 \right\rangle - \widetilde{\Gamma }(\varvec{\rho }_a,\varvec{\rho }_b)^2 . \end{aligned}$$

of the modified correlation function. Derivation of $\mathcal {F}(\varvec{\rho }_a,\varvec{\rho }_b)$ with respect to *K* immediately shows that the minimum is reached for

6 $$\begin{aligned} K = \bar{K}(\varvec{\rho }_b) = \frac{\langle \Delta I_b (\varvec{\rho }_b)\Delta I_a^{\mathrm {TOT}} \rangle }{\langle (\Delta I_a^{\mathrm {TOT}})^2\rangle }. \end{aligned}$$

The different outcome of the analysis when considering the standard correlation function of Eq. (1) and the modified one of Eq. (4) can be found in the Supplementary Information.

### Viewpoint multiplicity

The viewpoint multiplicity is defined as the effective number of viewpoints per transverse direction. In the case of CLM, the viewpoint multiplicity is estimated as the number of resolution cells falling within the diameter *D* of the objective lens. The resolution cell must be evaluated by considering that, in the correlation function, the sample acts as an aperture, and thus determines the resolution on the objective lens (see Ref. ^44^ for details). Considering a sample made of a bright object of diameter *a*, placed in the focal plane of the objective lens, the size of the resolution cell on the lens plane reads

7 $$\begin{aligned} \Delta x_{\mathrm {lens}} = 1.22\, \frac{\lambda f_O}{a}. \end{aligned}$$

Therefore, the viewpoint multiplicity can be evaluated as

8 $$\begin{aligned} \frac{D}{\Delta x_{\mathrm {lens}}} = \frac{D}{1.22\, \lambda f_O} a = \frac{a}{\Delta x_0}. \end{aligned}$$

This result highlights an interesting reciprocity relation between the two apertures and the two resolution cells, on the objective lens and the object plane. The above results, evaluated for an object in the focal plane, are still approximately valid if the object axial position *z* satisfies $|z-f_O|\ll f_O$.

### Axial resolution

The DOF of each single refocus image provides information on the CLM axial resolution. As reported in the second column of Table 1, CLM is characterizd by the same DOF as a standard microscope in the objective focal plane, determined by the numerical aperture and wavelength of illumination. When the object is moved out of focus, the DOF of the refocused image can be determined by a geometrical-optics approach. If two point sources, placed on opposite sides of the optical axis and separated by a distance *a* along the transverse direction *x*, are located on a transverse plane placed at a distance *z* from the objective, the correlation function they generate reads

9 $$\begin{aligned} \Gamma (\varvec{\rho }_a,\varvec{\rho }_b) \sim \left[ \delta ^{(2)} \! \left( - \frac{z}{f_T} \varvec{\rho }_a - \left( 1 - \frac{z}{f_O} \right) \frac{\varvec{\rho }_b}{M_L} -\frac{a}{2} \hat{u}_x \right) + \delta ^{(2)} \! \left( - \frac{z}{f_T} \varvec{\rho }_a - \left( 1 - \frac{z}{f_O} \right) \frac{\varvec{\rho }_b}{M_L} +\frac{a}{2} \hat{u}_x \right) \right] P^2 \left( - \frac{\varvec{\rho }_b}{M_L} \right) , \end{aligned}$$

as given by Eq. (2), with $\delta ^{(2)}(\varvec{\rho })$ the two-dimensional Dirac delta and $\hat{u}_x$ the unit vector along the *x*-axis. By refocusing the image described by Eq. (9) through the refocusing algorithm of Eq. (3), we see that, as soon as refocusing is implemented on an axial coordinate different from the one identifying the object position ($f\ne z$), each point-like source generates a “circle of confusion” analogous to the one of conventional imaging. Unlike other CLM features, the circle of confusion depends on the numerical aperture of the CLM device. The refocused image at a generic distance $f\ne z$ reads

10 $$\begin{aligned} \Sigma _{\mathrm {ref}} (\varvec{\rho }_a) = P^2\left( \frac{z}{f-z}\left( -\frac{\varvec{\rho _a}}{M} - \frac{f}{z} \frac{a}{2} \hat{u}_x \right) \right) + P^2\left( \frac{z}{f-z}\left( -\frac{\varvec{\rho _a}}{M} + \frac{f}{z} \frac{a}{2} \hat{u}_x \right) \right) . \end{aligned}$$

Assuming the objective lens transmission function *P* is a circular iris of radius *R*, Eq. (10) represents two circles of radius $MR|f-z|/z$ and centered in $\pm Mf/2z\,\varvec{a}$. Therefore, there will exist a range for which the refocusing parameter *f* generates two separate circles; outside of that range, the two circles begin to overlap and, ultimately, the two sources can no longer be resolved. Particularly, there will be two refocusing positions $f^\prime$ and $f^{\prime \prime }$ for which Eq. (10) describes two tangent circles. We thus define the DOF of the refocused images as

11 $$\begin{aligned} \Delta z =|f^{\prime }-f^{\prime \prime }|\simeq \frac{a}{\text {NA}_0}, \end{aligned}$$

where the approximation holds for $|f^{\prime}-f^{\prime\prime}|\ll f_O$. Hence, depending on the size of the object details of interest, the images refocused by CLM have a DOF that depends on the numerical aperture in the same fashion as for a standard microscope. However, the ratio between the extended DOF available to CLM and the DOF of the refocused image gives the number of independent planes accessible through refocusing, which is:

12 $$\begin{aligned} N_\text {CPI}=\frac{3.3\, a^2/\lambda }{a/\text {NA}_0}\simeq \frac{2a}{\Delta x_0}. \end{aligned}$$

This result shows that, as is the case of conventional light-field imaging, the number of longitudinal planes that can be refocused is proportional to the viewpoint multiplicity on the lens plane. The advantage of CLM over conventional light-field imaging is the larger accessible viewpint multiplicity, as already discussed in the “Introduction”.

## Supplementary Information

Supplementary Information.

## Data Availability

The datasets used and/or analysed during the current study are available from the corresponding author on reasonable request.
