# Supplementary material for: Light-field microscopy with correlated beams for high-resolution volumetric imaging
Source: Sci Rep. 2022 Oct 7;12:16823. doi: 10.1038/s41598-022-21240-1 (PMC9547068; doi:10.1038/s41598-022-21240-1)
Supplement: Supplementary file 1 — Supplementary Information. [file 41598_2022_21240_MOESM1_ESM.pdf]

SUPPLEMENTARY INFORMATION
Light-field microscopy with correlated beams for high-resolution volumetric imaging

Gianlorenzo Massaro,[1,2] Davide Giannella,[1,2] Alessio Scagliola,[1,2] Francesco Di Lena,[2]
Giuliano Scarcelli,[3] Augusto Garuccio,[1,2] Francesco V. Pepe,[1,2] and Milena D'Angelo[1,2]

[1]*Dipartimento Interuniversitario di Fisica, Università degli studi di Bari, I-70126 Bari, Italy*
[2]*INFN, Sezione di Bari, I-70126 Bari, Italy*
[3]*Fischell Department of Bioengineering, University of Maryland, College Park MD 20742, United States*

Figure 1 reports four cases of refocused images of a triple slit ($d = 44.2\,\mu$m, corresponding to point A' in Fig. 3(a) of the main text) placed outside the DOF of the conventional microscope ($f - f_O = 1.00\,$mm); CLM refocusing has been implemented by computing correlations and averaging over different numbers of acquired frames N, ranging from 1 to $25,000$. The image quality clearly improves as the number of frames increases from 100, where the image of the object begins to be observable, to 5×10^3, while improvements become negligible for larger number of frames. Since the sample is a binary object (*i.e.* made up of either transmissive or non-transmissive details), an estimation of the final image quality can be obtained by considering a signal-to-background ratio (SBR), where the image of transmissive parts of the object is considered as signal, and the remaining parts of the frame as background. We define the SBR by normalizing the refocused images from 0 to 1 and separating pixels in the two subsets

$$I_{\text{signal}} = \{(i,j) \text{ such that } \Sigma_{i,j} > 1/2\}, \quad (1)$$
$$I_{\text{background}} = \{(i,j) \text{ such that } \Sigma_{i,j} \leq 1/2\}, \quad (2)$$

with $\Sigma_{i,j}$ the normalized signal and (i,j) the indeces identifying pixel positions. The cardinalities $|I_{\text{signal}}|$ and $|I_{\text{background}}|$ are then used to compute the mean values of the signals in the two sets of data:

$$\overline{\Sigma}^{(\text{signal})} = \frac{1}{|I_{\text{signal}}|} \sum_{(i,j)\in I_{\text{signal}}} \Sigma_{i,j}, \quad (3)$$
$$\overline{\Sigma}^{(\text{background})} = \frac{1}{|I_{\text{background}}|} \sum_{(i,j)\in I_{\text{background}}} \Sigma_{i,j}, \quad (4)$$

from which we get:

$$\text{SBR} = \frac{\overline{\Sigma}^{(\text{signal})}}{\overline{\Sigma}^{(\text{background})}}. \quad (5)$$

The values of the SBR characterizing the CLM refocused images of Fig. 1, are reported in Table I for a number N of acquired frames ranging from 1 to $25,000$. Interestingly, the triple slit is already visible with $N = 100$, with a SBR = 3.2; this indicates an improvement of the SBR by one order of magnitude in the present scheme with respect to the original CPI scheme (see Ref. [23] of the main text). Though the theoretical expectation for true chaotic illumination supports a $\sqrt{N}$ scaling of the quantity reported in Table I, we observe an evident saturation effect after $N = 5,000$ frames. This is related with the fact that our chaotic source is based on a rotating ground-glass disk, hence, the patterns of chaotic light that illuminates the transmissive parts of the test target, in each frame, are available in a finite number and are not completely independent from each other.

N	SBR
1	1.0
10	1.7
100	3.2
1,000	4.2
2,000	4.6
5,000	5.7
10,000	5.8
25,000	6.2

TABLE I. Signal-to-background ratios (SBR) of the CLM refocused images of the triple slit masks reported in Fig. 1, computed from Eq. (5) by employing a varying number N of acquired frames.

Fig. 2 and 3 demonstrate the improved performances of CLM over a standard microscope, in terms of resolution and depth of focus. Fig. 2 shows the accordance between the theoretical resolution, estimated as the distance between two slits that can be refocused with 10% visibility, and the experimental one. In particular, we explore the range between −1 mm and +1 mm along the optical axis, in steps of 250 μm. The two solid lines in Fig. 2 panel (a) are the 10% visibility curves for standard microscopy (orange) and CLM (blue). Each pair of red dots, labeled as A to D, represent the center-to-center distance in triple-slit masks of a 1951 USAF resolution test target. Two resolutions have been tested for each different position of the target along the optical axis. At any given position on the optical axis, we employ the smallest element on the target having slit distance larger than the theoretical visibility curve (lower point in each pair) and the immediately larger element (upper point in the pair). Pairs labeled A' to D' are symmetrically displaced, with respect to the focused plane, to their non-primed counterparts. The comparison between the (unfocused) microscope images and the CLM refocused images is shown in Fig. 2 panel (b). Upper points in each pair are very clearly resolved, as prescribed by the theory. Points closer to the 10% theoretical visibility (i.e., lower points), on the other hand, are less resolved

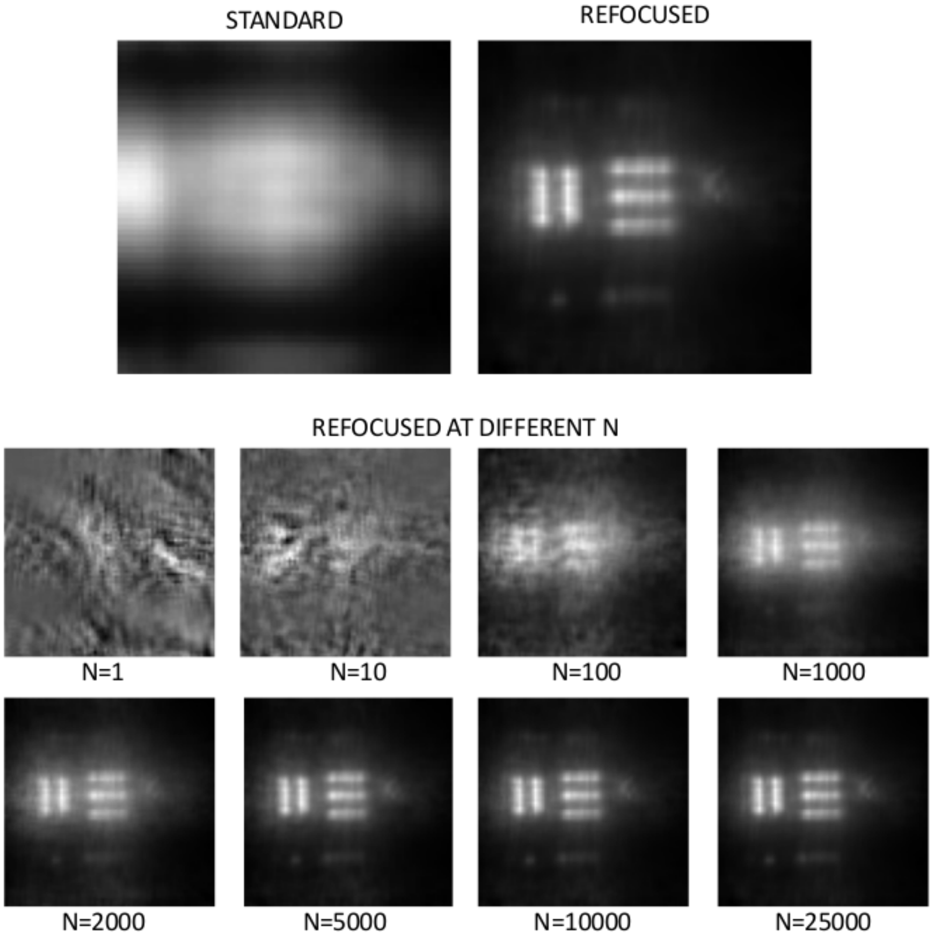

FIG. 1. *Upper panels.* Comparison between images of a triple slit ($d = 44.2\,\mu$m) placed out of focus ($f - f_O = 1.00$mm), as directly acquired by the microscope (left panel) and refocused by CLM (right panel). *Middle and lower panels.* Images of the same triple slit refocused by using a different number N of frames: middle panel, from left to right, $N = 1, 10, 100, 1000$, lower panel, from left to right: $N = 2000, 5000, 10000, 25000$.

and offer the chance to analyze the effect of the loss of resolution in CLM refocused images: All of them, in fact, show the typical fringes of coherent imaging. This is not unexpected, since CLM is by all means a coherent imaging technique, although based on an incoherent source of light [29]. The effect of the loss of resolution in CLM is also displayed on the right panel of Fig. 3, where elements 4, 5 and 6 of group 5 of the test target (corresponding to 22.1 μm, 19.7 μm and 17.5 μm resolutions) are visible. Element 4 is the lowest one in the image and corresponds to the upper point of pair D in Fig. 2. As the details become smaller, as in element 5 (lower point of pair D), fringes appear between the slits and keep degrading the image resolution to the point where they prevent the three slits from being distinguished (element 6). Point E in Fig. 2 is close to diffraction limit of a standard microscope and shows that CLM is capable of the same resolution at focus of a conventional microscope with the same NA. However, since the optics and magnifications have been chosen having in mind imaging of samples with cell-like details (i.e., tens of microns), the diffraction-limited resolution of the microscope cannot be reached due to the size of the pixels; in fact, with our magnification of 4.2 and pixel size of $6.5\,\mu$m (see Material and Methods), adjacent resolution cells end up in the same pixel. The left side of Fig. 3 shows the comparison between the microscope and CLM images in the focused plane. The last four elements of group 7 of the test target are perfectly resolved to the minimum available slit distance of $4.4\,\mu$m. Also, as one would expect from the visibility curves, the resolution is symmetric with respect to the focused plane.

As mentioned in the Material and Methods section of the main article (“Noise mitigation in the CLM correlation function” subsection), we have developed a novel approach to data analysis in CPI, aimed at improving its SNR performances. Here we show that this technique helps getting rid of the spurious background coming from out-of-focus planes within the refocused CLM images of complex three-dimensional samples. In Fig. 4, we com-

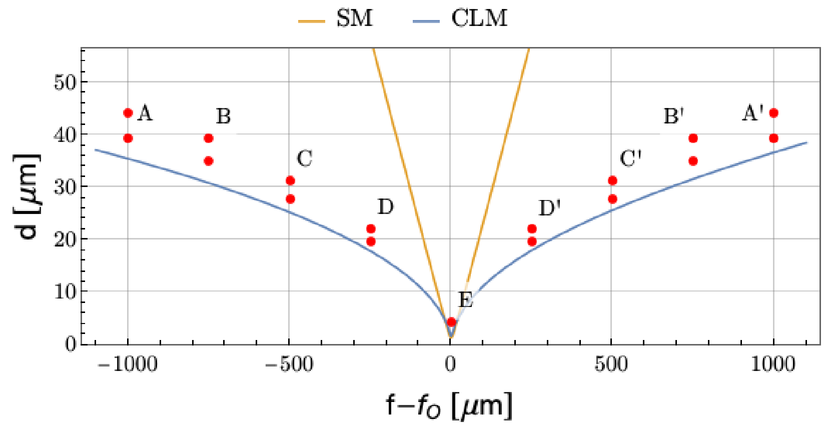

(a)

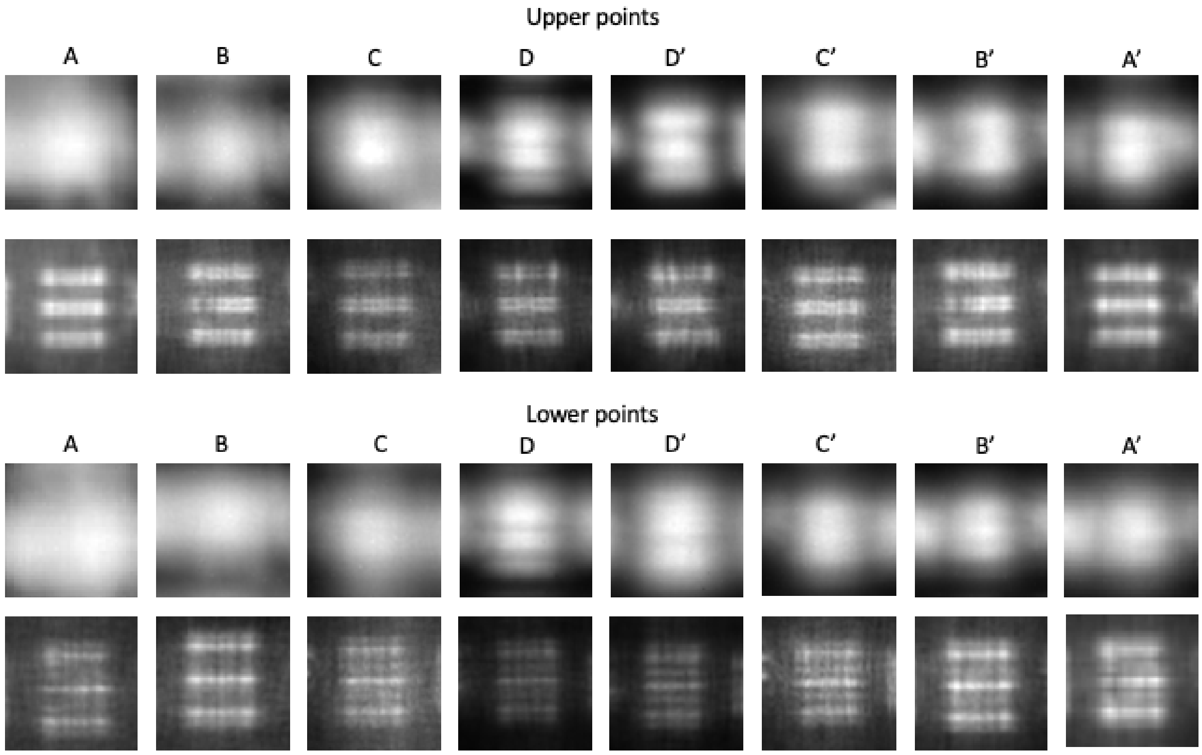

(b)

	A	B	C	D	D'	C'	B'	A'
Displacements $(f - f_O)$ [μm]	-1000	-750	-500	-250	250	500	750	1000
(d) [μm] for upper points	44.2	39.4	31.3	22.1	22.1	31.3	39.4	44.2
(d) [μm] for lower points	39.4	35.1	27.8	19.7	19.7	27.8	35.1	39.4

(c)

FIG. 2. Demonstration of the resolution versus DOF improvement of CLM, as the object (a resolution test target) is moved away from the focused plane. *Panel* (a): 10%-visibility limits in standard imaging (solid line, orange) and CLM (solid line, blue), as reported in Fig. 3(a) of the main text, as a function of the longitudinal displacement $f - f_O$ from the objective focal plane. The pairs of red dots labeled A to D represent the combinations of center-to-center slit distance d and displacement $f - f_O$ at which the experimental data reported in panel (b) have been acquired, as detailed in table (c). Points labeled with a primed letter are placed symmetrically with respect to the focused plane to their non-primed counterpart. Point E is an acquisition with the target in the focused plane, and the corresponding acquired image is shown in Fig. 3 (left bottom panel). *Panel* (b): the upper (lower) part of the panel shows the comparison between the unfocused micrsocope (SM) images and the CLM refocused images for the each element of the upper (lower) series of points indicated in panel (a). The table in *panel* (c) reports the values of $f - f_O$ and d related with the different employed samples.

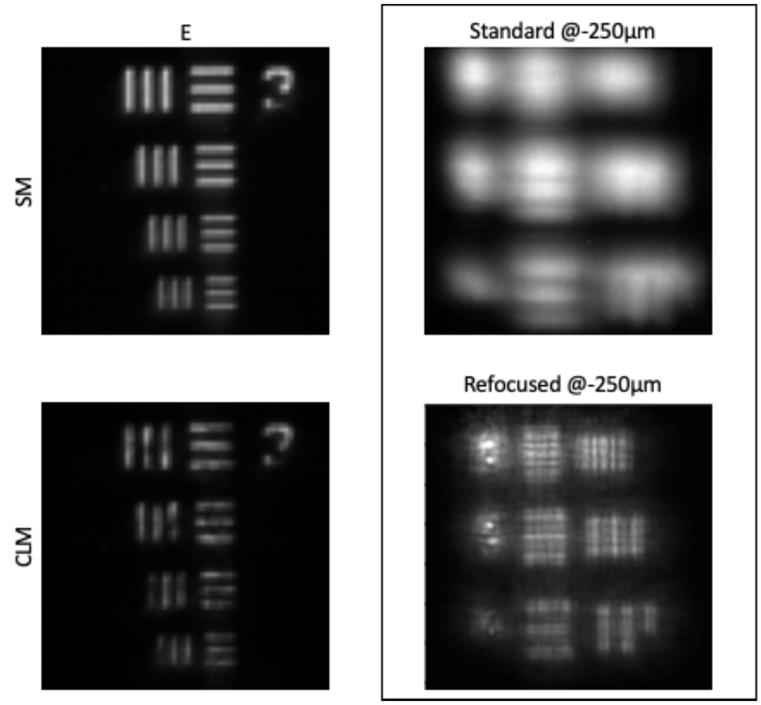

FIG. 3. *Left*: comparison between the standard microscope (SM) and CLM images in the focused plane. The four smaller elements of group 7 of the resolution test target are imaged ($6.2\,\mu$m, $5.5\,\mu$m, $4.9\,\mu$m, and $4.4\,\mu$m) by CLM with the same resolution as in a conventional microscope. *Right*: microscope and refocused image of elements 4, 5, 6 (from bottom to top) of group 5 of the test target ($22.1\,\mu$m, $19.7\,\mu$m, $17.5\,\mu$m), placed out of focus at a longitudinal distance $f - f_O = -250\,\mu$m, which is very close to the 10%-visibility curve of CLM. In the CLM image, as the separation between neihghboring slits decreases, fringes due to interference appear and the slits can no longer be resolved. The appearance of non-resolved details is very different from that typical of standard imaging (upper image) due to the intrinsic coherent nature of CLM.

pare the results obtained by refocusing a complex thick three-dimensional sample through the correlation function of Eq. (1) of the main text (same data employed to get Figs. 4-5 of the main text) with the ones obtained by the new approach through minimization of the fluctuations. This approach clearly improves the quality of the CLM refocused images by cleaning up the typical background due to out-of-focus planes within the three-dimensional sample.

Fig. 5 shows that CLM enables the 3D localization of a single starch granule within the volume occupied by the sample. In the upper image, we report the result of refocusing on the axial plane $z = 200\,\mu$m, as already shown in the main text and in Fig. 4 of this document. From the image, a single starch granule can be identified at coordinates ($37\,\mu$m, $210\,\mu$m). The resolution on this plane is the same along x and y and depends on the axial coordinate on which refocusing occurs (see Table 1 in main text). Given the experimental parameters, the transverse resolution at $z = 200\,\mu$m is estimated as $\delta x = \delta y = 11.4\,\mu$m. The two lower panels show the xz section (left) and yz section (right) of the volume at y and x coordinates fixed by the position of the refocused granule. Unlike axial planes, however, coronal and sagittal planes are not available with the same resolution throughout their extension: in fact, Table 1 in the main text prescribes a degradation of both axial and transverse resolution with the axial distance from the focal plane of the objective lens. Moreover, the axial resolution δz is always expected to be worse than the transverse resolution, since $\delta z = \delta x/\mathrm{NA}$ at any given axial position. Hence, both in sagittal and coronal planes, the resolution cell is expected to be rectangular, instead of squared, and its area increases with the distance from the plane at focus. The theoretical axial resolution on the refocused position is $\delta z = 47.0\,\mu$m. In the lower panels of Fig. 5, the voxels related to two more axial coordinates are also reported, corresponding to $z = 100\,\mu$m ($\delta x = 8.1\,\mu$m and $\delta z = 33.0\,\mu$m), and $z = 300\,\mu$m ($\delta x = 14.0\,\mu$m and $\delta z = 57.2\,\mu$m).

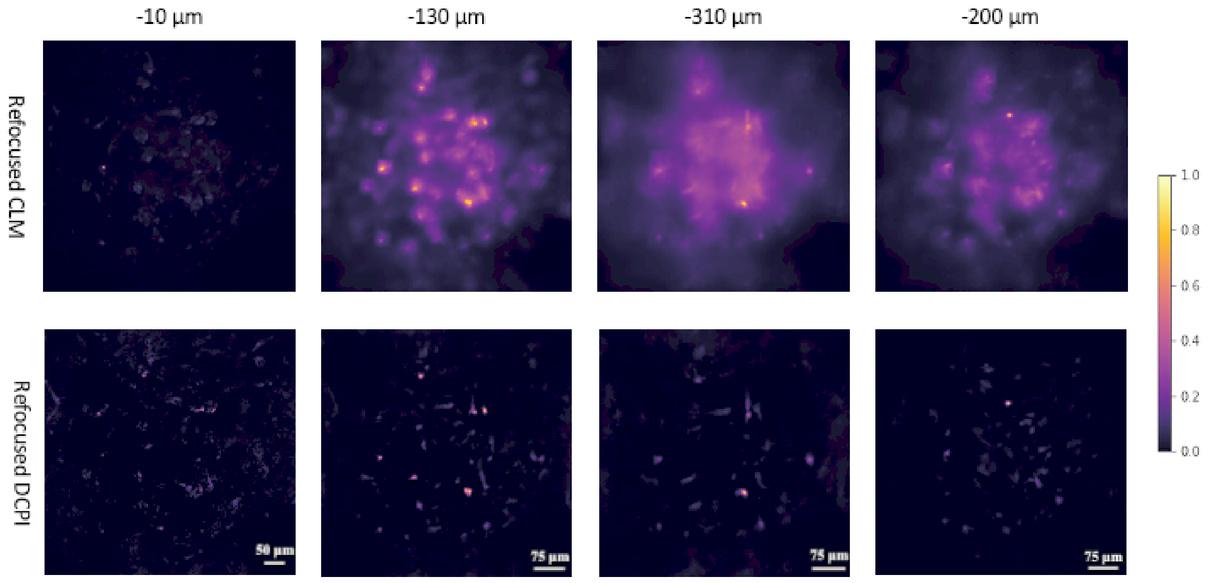

FIG. 4. CLM refocused images of four distinct planes inside a starch dispersion in gel (upper panels) and the corresponding images obtained by applying the noise minimization algorithm described in the “Materials and Methods” section (lower panels). All images were elaborated from the same acquisition of the correlation function using $N = 5000$ acquired frames, as in the main text. Data in the bottom row are the same reported in Fig. 4 of the main text, but in a different color scale that makes background suppression more evident.

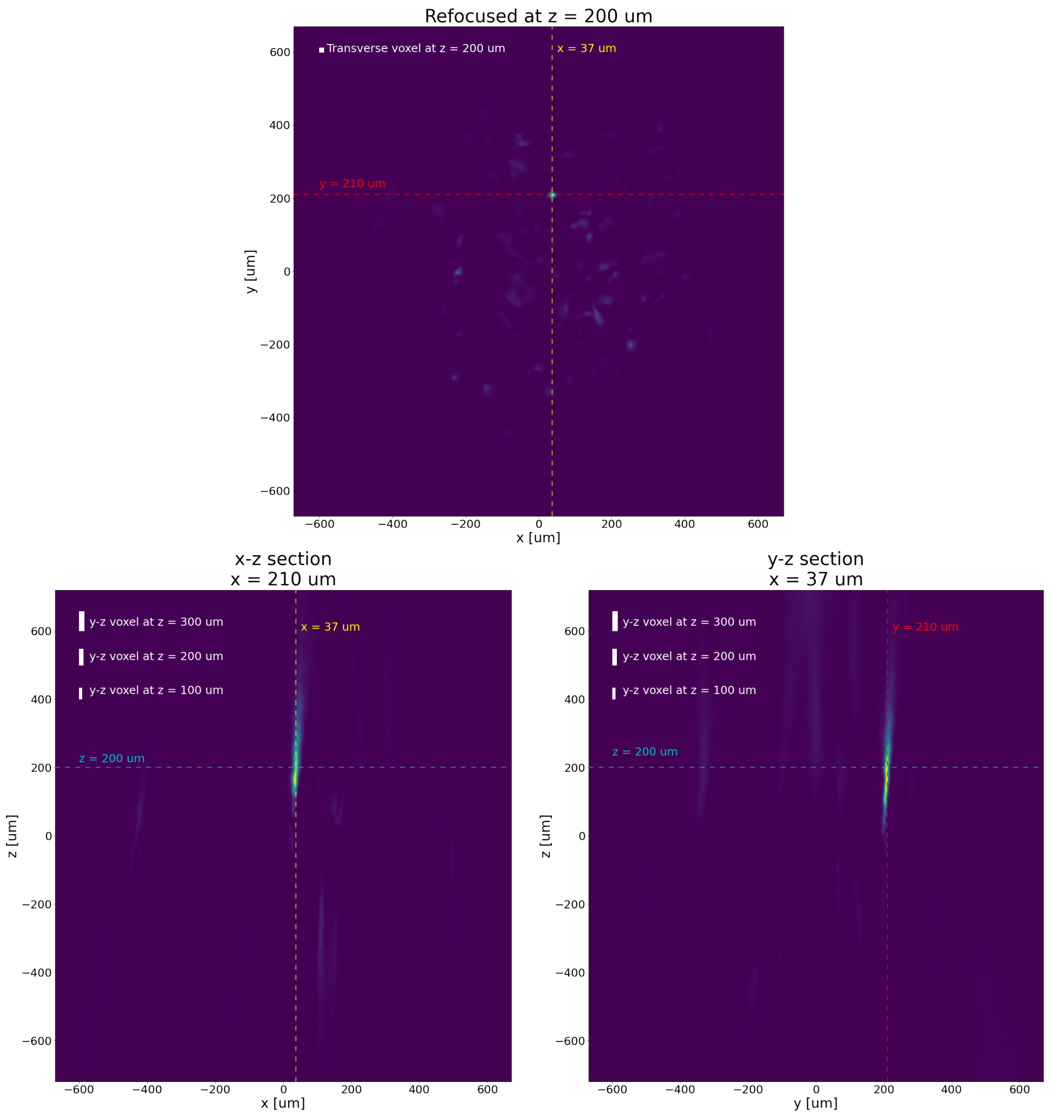

FIG. 5. Refocused image (top) of the starch dispersion in gel, corresponding to a xy-sectioning of the sample at axial coordinate $z = 200\,\mu$m. Lower panels show the xz (left) and yz (right) slices centered on the starch granule that is visible in the refocused image.
